# Exploring Emotional Intelligence and Sociodemographics in Higher Education; the Imperative for Skills and Curriculum Development

**DOI:** 10.3390/bs13110911

**Published:** 2023-11-08

**Authors:** Sanaa Abouhasera, Marawan Abu-Madi, Mohammed Al-Hamdani, Atiyeh M. Abdallah

**Affiliations:** 1 Department of Biomedical Sciences, College of Health Sciences, QU Health, Qatar University, Doha 2713, Qatar; 200360548@student.qu.edu.qa (S.A.); abumadi@qu.edu.qa (M.A.-M.); 2 Division of Transfusion Medicine, Hamad Medical Corporation, Doha 3050, Qatar; 3 Department of Public Health, College of Health Sciences, QU Health, Qatar University, Doha 2713, Qatar; malhamdani@qu.edu.qa

**Keywords:** emotional intelligence, job and academic performance, age, gender, marital status, TEIQue-SF, Qatar

## Abstract

There is increasing interest in understanding the nature and impact of emotional intelligence (EI) in educational institutions and the workplace since EI is associated with academic performance, career success, job satisfaction, and management skills. Here we measured EI levels in students and employees at Qatar University and examined associations with sociodemographic variables. This cross-sectional study used the Trait Emotional Intelligence Questionnaire-Short Form (TEIQue-SF) questionnaire to quantify EI. Of 517 respondents, 315 were students and 202 were employees. The mean global EI level across all participants was 4.80 ± 0.78, with EI highest in the well-being domain (5.43 ± 1.04). Overall, older respondents had higher EI than younger respondents. There was no significant effect of gender, marital status, or employment position on EI. However, there were significant two- and three-way interactions. As a standalone variable, age was the most important factor influencing EI development in our cohort. However, three-way interactions revealed complex effects between age, gender, and marital status and EI. Our findings support a need for workshops on EI for employees and integrating dedicated courses into existing curricula to equip students with effective interpersonal relationship skills that foster EI development. Developing such interpersonal skills could help to promote personal, professional, and academic success.

## 1. Introduction

Emotional intelligence (EI) describes the capacity to observe and differentiate one’s own emotions and those of others and apply this knowledge to direct one’s thoughts and behaviors [1]. EI encompasses a constellation of personal emotional perceptions located at the lower levels of personality hierarchies that combine the emotional features of personality [2]. EI has recently attracted interest due to its ability to predict a set of characteristics associated with better social interactions, a higher prospect of attaining goals, and successful people management skills [3,4]. The term was initially introduced in 1990 by Salovey and Mayer, who defined it as “the ability to monitor one’s own and others’ emotions, to discriminate among them, and to use the information to guide one’s thinking and actions” [5]. Since its inception, the concept of EI has gained a significant amount of academic attention [6,7]. Initially, the concept of EI was developed within the field of psychology but, as research on the topic progressed, the concept expanded to other fields such as education, leadership and management, human resources, and career development. However, robust evidence that training emotional competencies improves job and academic performance is still lacking [8].

There are three main EI models: trait, ability, and mixed [9]. The ability model proposes EI as a narrow set of objectively measurable, interrelated cognitive emotional abilities that involve the ability to recognize, control, encourage, and interpret the emotions of oneself and others [10]. Conversely, trait EI, first presented by Petrides in 2001, refers to an individual’s self-perceptions of their own emotional capabilities. Trait EI defines our viewpoint on our emotional experiences, therefore reflecting our emotional tendencies and our perception of how skilled we are at recognizing, comprehending, regulating, and employing our own and other individuals’ emotions [11]. Trait EI addresses “emotional self-projections” placed at the lower levels of personality structure and incorporates the “affective aspects” of human personality [12]. Trait EI therefore combines emotional personality features and is largely separate from human cognitive capacity. The mixed model is made up of two main models, the trait and the ability [9].

EI has been linked to career success and successful management, and it has been shown to have a direct influence on job satisfaction, workplace stress, and employee efficiency. In addition, it influences academic buoyancy and student engagement [13] and enhances student retention in higher education [14]. Moreover, EI is a critical skill in problem-solving, decision-making, and conflict resolution [15], and it can promote strong ethical values and positive manners in the workplace [16]. Individuals with high EI can usually regulate their emotions, think critically, and communicate effectively, ultimately leading to better outcomes and a significant positive impact on organizational performance. In the workplace, effective emotional management when interacting with others is important for creating a healthy working culture, helping employees to be more emotionally self-aware and expressive and to understand the feelings of others. In addition, EI increases their tolerance, integrity, trust, and productivity by improving work relationships within the organization. EI enables leaders to regulate and manage their emotions, communicate efficiently, and coordinate with others in the workplace to achieve common goals, promote empathy, and participate in effective group activities. Additionally, it enhances leaders’ ability to understand others and establish better working relationships with co-workers. It has been shown that EI in leadership is important for achieving success, commitment, and positive outcomes [17]. Given this context, leaders must shape policies and programs to strengthen their employees’ EI levels, since this would be expected to benefit the organization.

Therefore, educational institutions should recognize the importance of EI for their employees and students. Understanding EI across the workforce and student body in higher education institutions could improve efficiency at every level: in students, for growth and development; in staff, for productivity and management. The Trait Emotional Intelligence Questionnaire (TEIQue), developed by University College London, is a freely accessible, self-reported questionnaire used to assess the facets of trait EI based on sampling domains that seek to frame the emotional features of personality. TEIQue is generally preferred over other questionnaires measuring EI because it offers a comprehensive representation of all trait EI sampling domains and has high predictive accuracy [2]. It is also widely used and has been translated into over 20 languages. However, there have been no previous studies of EI in Qatar, even though population-specific data are important since the sociodemographic variables associated with EI vary according to geography, studied group, and assessment approach. Here we aimed to assess EI levels in a representative education sector population in Qatar (employees and students at Qatar University (QU)). We also examined associations between sociodemographic variables and EI in this population. The aim of this study was to answer two research questions. (Q1) Do age, gender, position, and marital status individually affect global EI? And, (Q2) do age, gender, position, and marital status have interactive effects on global EI?

## 2. Materials and Methods

### 2.1. Ethical Approval

The Institutional Review Board of QU approved the study (reference number QU-IRB 1731-E/22). Participation was voluntary, and data collection was anonymous. All information and records generated in this study were strictly confidential. Electronic informed consent was obtained from participants before starting the questionnaire.

### 2.2. Design

A 2 age (younger or older adults) × 2 gender (male or female) × 3 position (administration, faculty, or student) × 2 marital status (single or married) design was used in this study. The outcome of interest was total EI scores.

### 2.3. Population and Sampling

The target subjects were students, faculty, and administrators at QU. All QU students in any program and at any level, as well as academic or administration employees, were eligible to participate. All participants were English or Arabic speakers and were over 18 years old. Data were collected between September and October 2022. Participants were recruited through a questionnaire link provided in e-mail announcements. The questionnaire was built and administered using Google Forms.

### 2.4. Procedure and Instrument

**Figure 1:**
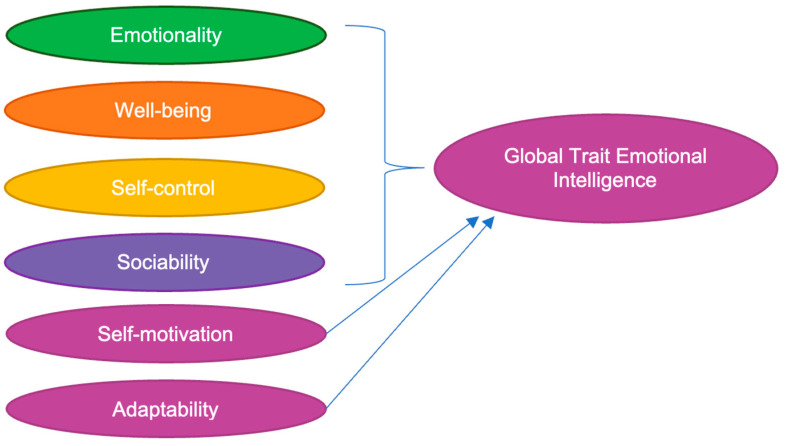
Trait EI is assessed through fifteen distinct facets of emotion categorized into four domains: emotionality, sociability, well-being, and self-control. These domains take thirteen of the facets into account, while the remaining two directly contribute to the global trait EI score [19].

Trait EI is usually assessed through fifteen distinct facets of emotion categorized into four domains: emotionality, sociability, well-being, and self-control. These domains take thirteen of the emotional facets into account, while the remaining two (self-motivation and adaptability) directly contribute to the global trait EI score (Figure 1). Emotionality is related to the expression and perception of emotions, while sociability is related to social connections, interactions, and the impact of an individual in their social atmosphere. Well-being is related to an overall sense of comfort, happiness, and contentment, whereas self-control is related to control over desires and urges [18].

This study used the TEIQue-Short Form (TEIQue-SF), a summarized form of the TEIQue. TEIQue-SF is a 30-item questionnaire based on trait EI theory that provides a comprehensive analysis of the four trait EI domains of emotionality, sociality, well-being, and self-control. The TEIQue-SF uses a Likert-style response format ranging from 1 (completely disagree) to 7 (completely agree). The TEIQue is a validated, publicly available instrument intended for academic or medical research use and it is available on the London Psychometric Laboratory website (https://psychometriclab.com/obtaining-the-teique/ accessed on 1 February 2022). Participants needed ~15 min to complete the questionnaire, which was offered in English and Arabic, the latter validated previously [20]. The short form has the advantage of being usable when time is limited.

The TEIQue-SF questions ensure comprehensive coverage of the sampling domain of trait EI (see Supplementary Tables S1 and S2). Briefly, emotionality (assessed by questions 1, 2, 8, 13, 16, 17, 23, and 28) is related to the expression and perception of emotions as well as using those attributes to build, improve, and maintain personal relationships with loved ones. People who score highly on this factor can recognize their internal emotions and are able to express their feelings. Well-being (assessed by questions 5, 9, 12, 20, 24, and 27) relates to an overall sense of comfort, happiness, and feeling well. People who score high in this factor are expected to be happy, positive, optimistic, and content. By contrast, individuals with low scores are more likely to be disappointed, with poor self-esteem and dissatisfaction with their lives. Self-control (assessed by questions 4, 7, 15, 19, 22, and 30) relates to the extent of control over desires and urges. Individuals who score high in this domain can control impulses and regulate stress, while individuals with low scores are susceptible to reckless behavior and may struggle to control external pressures. Finally, sociability (assessed by questions 6, 10, 11, 21, 25, and 26), unlike the emotional factor above, highlights social connections, interactions, and impact; that is, it considers the role of an individual in their social atmosphere. People with high sociability scores are good at communication, good listeners, good negotiators, and can affect other’s emotions. Conversely, people with low scores often appear reserved and shy. Adaptability and self-motivation fall under global trait EI and are assessed by four questions (3, 14, 18, and 29). Finally, basic sociodemographic data were collected from participants including age, gender, position, and marital status.

### 2.5. Data Analysis

The data obtained from the questionnaire were tabulated in Excel and results were entered into the TEIQue scoring engine available on the London Psychometric Laboratory website (https://psychometriclab.com/scoring-the-teique/ accessed on 1 February 2022) for interpretation. All statistical analyses were performed in SPSS v.29 (IBM Statistics, Armonk, NY, USA). Data were analyzed by 4-way ANOVA with between-subject variables of age, gender, position, and marital status and one dependent variable of total EI score. Univariate effects were examined, and significant interactions were analyzed using simple effect least significant difference (LSD) tests. The sum of squares error IV was selected for unbalanced cell sizes because there were no missing data. The significance level was set at a *p*-value ≤ 0.05. If an interaction was significant, the simple main effects differences were assessed such that group differences in each layer of significant simple main effects were compared.

## 3. Results

### 3.1. Study Population

Five hundred and seventeen responses were collected from QU students and employees: 202 (39.1%) employees and 315 (60.9%) students enrolled in different programs at QU (Table 1). The majority were female (72.7%), 67.5% were single, 48.9% were between 18 and 24 years of age, and 36.6% were Qatari nationals. The mean global EI score was 4.80 ± 0.78. EI was highest in the well-being domain (5.43 ± 1.04), with emotionality and sociability slightly lower at 4.78 ± 1.03 and 4.71 ± 1.08, respectively. The lowest EI score was in the observed self-control domain (4.44 ± 1.02).

### 3.2. Univariable Analysis

In an unadjusted univariable analysis, there were significant associations between participant age and the well-being, self-control, and emotionality domains and global EI (Table 2), with particularly low scores in the 18–24 and 25–34 age groups. Gender was not associated with any domain. Marital status was associated with emotionality and well-being domains and global EI. Work status (i.e., student, administration, or faculty staff) was associated with the self-control and emotionality domains and global EI.

The adjusted univariable analysis of interactions between age, gender, position, and marital status is shown in Table 3. Age was the only variable that had a significant effect on global EI scores, with older individuals (M = 5.10, SE = 0.10) having higher scores than younger individuals (M = 4.78, SE = 0.07; *p* = 0.018). Although there were significant two-way interaction effects between gender and position and position and marital status, three-way higher-order interaction effects were also significant: age, gender, and position; age, gender, and marital status; and gender, position, and marital status.

**Figure 2:**
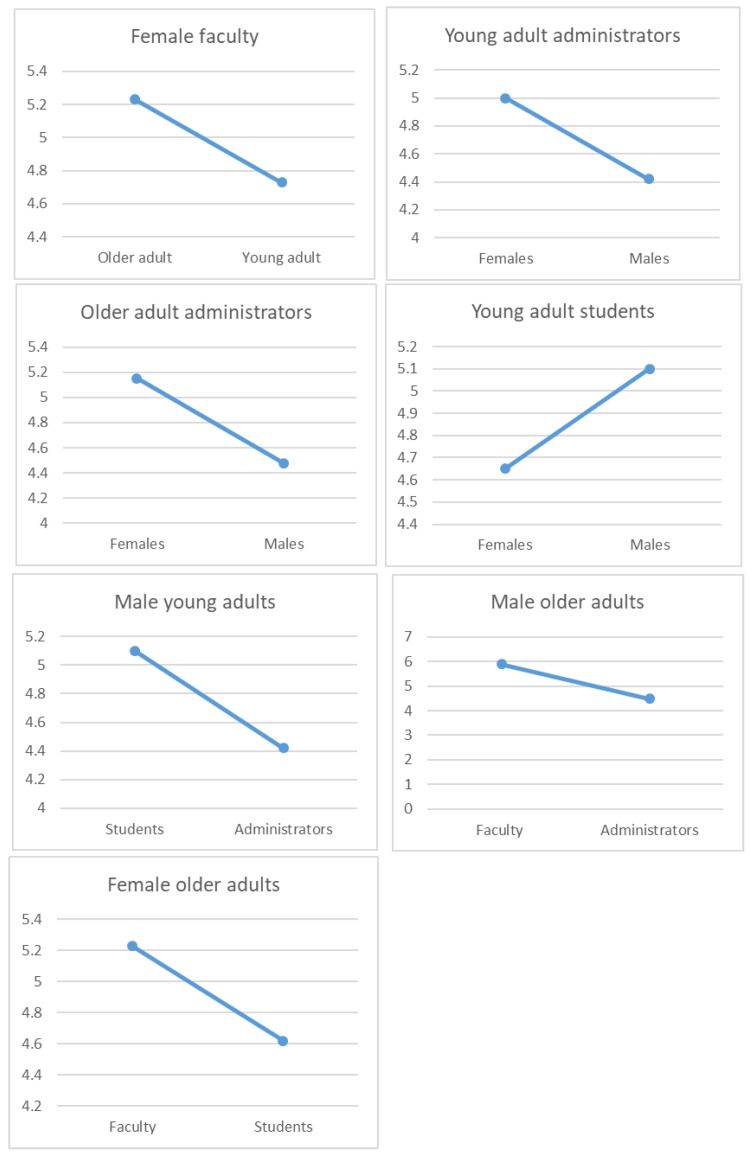
Significant pairwise differences in the age × gender × position comparison.

Examining the three-way interaction between age, gender, and employment position revealed several pairwise differences (Figure 2). Among female faculty, older individuals (M = 5.23, SE = 0.12) had higher global EI scores than younger individuals (M = 4.73, SE = 0.15; *p* = 0.011). Among young administrative staff, females (M = 5.00, SE = 0.13) had higher global EI than males (M = 4.42, SE = 0.18, *p* = 0.012), but the young female students (M = 4.65, SE = 0.07) had lower global EI scores than young male students (M = 5.10, SE = 0.17; *p* = 0.019). Among older administrative staff, females (M = 5.15, SE = 0.16) had higher global EI scores than their male counterparts (M = 4.48, SE = 0.27; *p* = 0.040), while young male students (M = 5.10, SE = 0.17) had higher global EI scores than administrative staff (M = 4.42, SE = 0.18; *p* = 0.025). Older male faculty (M = 5.89, SE = 0.38) had higher global EI scores than administrative staff (M = 4.48, SE = 0.18; *p* = 0.009), while older female faculty (M = 5.23, SE = 0.12) had higher global EI scores than students (M = 4.62, SE = 0.21; *p* = 0.044).

**Figure 3:**
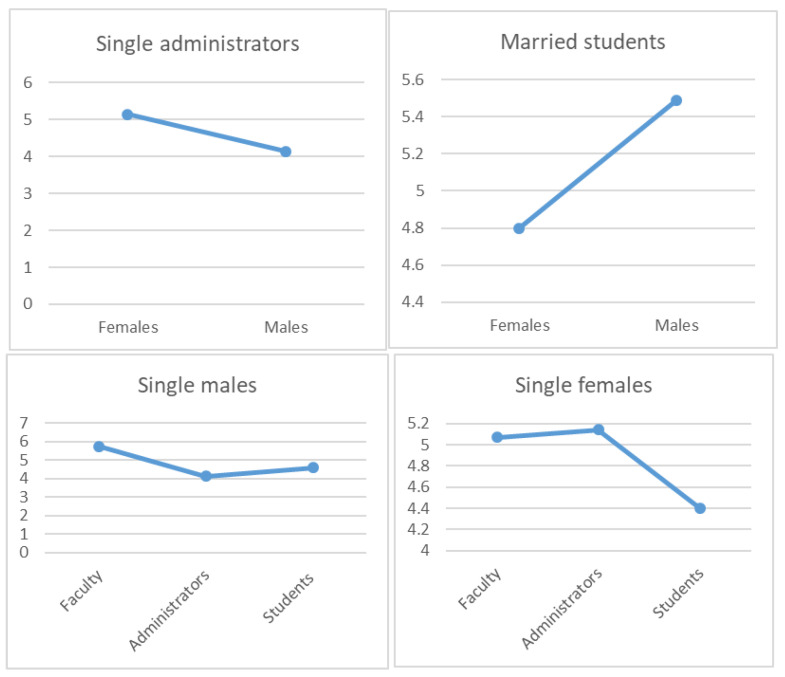
Significant pairwise differences in the gender × position × marital status comparison.

Similarly, there were many pairwise differences in the three-way interaction analysis of gender, employment position, and marital status (Figure 3). Among single administrative staff, females (M = 5.14, SE = 0.15) had higher global EI scores than males (M = 4.14, SE = 0.30; *p* = 0.003). Among married students, males (M = 5.49, SE = 0.27) had higher global EI scores than females (M = 4.80, SE = 0.13; *p* = 0.026). Among single males, faculty (M = 5.73, SE = 0.46) had higher global EI scores than both administrative staff (M = 4.14, SE = 0.30; *p* = 0.013) and students (M = 4.59, SE = 0.09; *p* = 0.048). Among single females, administrative staff (M = 5.14, SE = 0.15) had higher global EI scores than students (M = 4.4, SE = 0.19; *p* = 0.16), as did faculty (M = 5.07, SE = 0.13) relative to students (*p* = 0.029). No pairwise interaction between age, gender, and marital status interaction was significant.

## 4. Discussion

Here we performed a survey of EI in QU affiliates. The mean global EI score was 4.8 ± 0.78, consistent with previous reports (range 4.09 ± 0.77 to 5.11 ± 0.89) [20,21,22,23,24], and we confirmed that EI was significantly associated with age. Understanding EI and its nature in different age groups is crucial and likely to be determined by life and work experiences: global EI and the well-being, self-control, and emotionality EI domains were lower in younger adults, consistent with the hypothesis that EI is a developing skill that is likely to accumulate with life experience and added knowledge together with some abilities that must be developed through training.

Analysis of interactions between age, gender, and employment position revealed some interesting findings. Young female students scored lower than males. This suggests that global EI scores are position- and sex-dependent and may indicate that females entering the workforce develop higher global EI than males. This is consistent with some previous data on gender differences in student global EI scores [2], although others found no differences [25] and some have even reported higher scores in females than in males [26]. Further work is needed to examine the contextual factors that explain different patterns of EI scores between male and female students. In practice, it may be necessary to facilitate learning environments that bridge potential gaps in EI scores between males and females. Older male faculty had higher global scores than administrative staff, and older female faculty had higher scores than students, consistent with a pattern of higher EI among older faculty. It is possible that faculty positions attract individuals who possess high EI or, alternatively, working as faculty may help to develop EI.

Analysis of interactions between gender, position, and marital status also yielded interesting findings. Single female administrators scored higher than males, yet married male students scored higher than females, possibly indicating that marriage enhances EI scores in male students relative to female students [27], although the interaction needs further study. For both single males and females, faculty had higher scores relative to other positions. Longitudinal studies are needed to better understand the development and enhancement of EI among faculty prior to and during their tenure.

Both gender and age have been well studied in relation to EI, and several studies have reported associations between EI and age [28]. A relatively large study examined the relationship between EI and age in 6369 male and female participants aged between 18 and 65 years [29]. For both genders, EI increased steadily with age from 18 to 64 years, after which it decreased. A study of 357 Japanese medical students found that female participants tended to communicate emotions and were more competent with interpersonal expression than males, but there were no significant gender differences with respect to EI [30]. Although EI may be influenced by culture and gender, EI can be enhanced through education. Similarly, in an exploration of associations between EI, job performance, and social accountability in 270 healthcare professionals and caregivers aged between 27 and 42 years of age, total EI was significantly positively correlated with age, albeit across a narrow young age band [31]. Similar results were found in a study of 360 Chinese adults aged between 20 and 79 years [28]. Several studies have therefore shown that EI can increase with age and females show a higher level of EI than men [32]. EI is a skill that can be developed and improved with age and life experience. As individuals face challenges and overcome difficulties throughout their lives, they can acquire EI skills and manage their emotions more effectively. When individuals gain more exposure to different situations and learn to navigate complex life events with ongoing growth, individuals can continue to improve their EI over time. This explains why older people have higher EI levels than younger adults. However, these findings must be interpreted with caution, as EI is a complicated concept affected by many social and cultural aspects rather than just demographic features.

It is often believed that women have greater EI than men, and a few studies have supported this assumption [33,34,35]. This phenomenon might be because female relationships with parents, friends, and siblings tend to be more sensitive and stable, facilitating the development of EI [36]. On the other hand, several studies have reported the opposite, namely that males have a higher EI than females [37,38,39,40,41]. In our study, we detected no differences in global EI or subdomain scores between males and females, perhaps because personality and emotionality can be influenced by the person’s motivations, culture, social environment, and education. It is also possible that men and women merely express their emotions differently, leading to similar overall EI [42]. Our findings are consistent with a study from the United Kingdom, which found no significant association between gender and overall EI in a sample of employees [43]. Similarly, EI was not related to gender in other studies conducted in Egypt [42] and the USA [44,45] nor in undergraduate students in two other studies [36,46].

Our analysis showed that young adults, which included both students and young employees, had the lowest EI levels compared with other participants. This indicates a need to develop EI skills in young adults. One study reported that training nursing students in problem-solving skills could effectively enhance their EI [47]. To increase EI, it will be important to provide individuals with opportunities to develop emotional awareness and emotion regulation skills, perhaps through specific workshops, materials, and programs in the curricula. These may include teaching students how to identify, understand, and regulate their emotions, as well as how to empathize with others and handle interpersonal relationships effectively. Mentors can also demonstrate EI in their interactions with students and encourage a classroom culture that values emotional awareness and self-reflection. EI is a critical skill for students to develop as it can have a significant impact on their academic performance and social, personal, and professional growth. By developing their EI, students can recognize their emotions and manage them effectively, thereby improving their self-control, mental health, and problem-solving abilities. Additionally, students with higher EI can better understand and empathize with their peers, leading to improved social relationships and greater teamwork skills. Some EI domains were linked to marital status, suggesting a higher level of happiness and life stability with married people than with singles who possess low satisfaction and self-esteem. Older age married adults with higher educational levels are more exposed to life challenges and relationships, thus expressing more emotionality [48].

Several teaching techniques are available to achieve EI-related learning goals, but at the moment these are heterogeneous in terms of approach, coverage, and target learner. They can be as simple as low-cost online modules or as complex and costly as executive coaching. Moreover, the topics discussed in EI lectures may differ, with some emphasizing mindfulness, burnout, or resilience [49]. One study from Norway used storytelling as a method for teaching EI, which appeared to benefit students’ reflections on their role in the workplace. Those students exhibiting high EI tended to experience lower levels of stress [50]. Another study used a curriculum containing a variety of self-assessments and activities focusing on EI topics, and EI was assessed before and after program implementation; final scores were higher than the initial scores [51]. A similar study from Spain showed that students receiving an EI training program had enhanced life satisfaction and creativity, regardless of gender, compared with those who did not go through training [52]. In summary, to increase EI in students, it is important to integrate EI into the curriculum and provide opportunities for students to practice and reflect on their emotions and reactions. An assessment of the feasibility of online, in-person or combination workshops or the incorporation of EI-related learning goals in university curricula may enhance EI among young adults.

This study has some limitations. The response rate to the survey was low, and there was an unequal representation of male and female participants which may have introduced bias in our analysis. The survey-based study design, while widely used and valuable for gathering information, also has limitations, including the presence of self-selection bias. Given the select nature of the variables examined, we may have neglected other relevant factors that might have influenced the outcomes being measured. This may have limited the comprehensiveness of the findings and potentially led to incomplete or biased conclusions. The design and wording of the survey questions may have unintentionally introduced bias, leading to inaccurate or misleading responses.

## 5. Conclusions

Age as an individual variable was the only demographic variable that was associated with an increase in global EI. However, we detected a number of significant interactions among age, gender, position, and marital status, which convey the message that a number of demographic variables are associated with EI, in complex ways that require tailored training approaches to groups with lower EI levels. More training to bridge EI gaps, especially in different demographic groups, might be useful for enhancing EI at the institutional level. Future studies should focus on long-term longitudinal studies that follow career and life transitions would provide insights into the reasons behind changing EI scores. In addition, future research is needed to explore the relationship between EI and job performance together with its influence on students’ academic achievements, such as their grade point average.

## Figures and Tables

**Table 1:** Sociodemographic characteristics of the study respondents.

Variables	Number (%)	Variables	Number (%)
Gender		Position	
Male	141 (27.3)	Administrative staff	98 (19)
Female	376 (72.7)	Academic staff	104 (20.1)
Age		Student	315 (60.9)
18–34	380 (73.5)	Marital Status	
35 and over	137 (26.5)	Single	349 (67.5)
Citizenship		Married	168 (32.5)
Qatari	189 (36.6)		
Non-Qatari	328 (63.4)		

**Table 2:** Associations between all EI domain scores (mean and SD) with sociodemographic characteristics.

Variables	Well-Being		Self-Control		Emotionality		Sociability		Global EI	
	Mean ± SD	*p*-Value	Mean ± SD	*p*-Value	Mean ± SD	*p*-Value	Mean ± SD	*p*-Value	Mean ± SD	*p*-Value
Gender										
Male	5.3 ± 1.01	0.071	4.6 ± 1.0	0.023	4.8 ± 0.97	0.8	4.7 ± 1.01	0.6	4.8 ± 0.75	0.473
Female	5.5 ± 1.05		4.4 ± 1.02		4.8 ± 1.05		4.7 ± 1.10		4.8 ± 0.80	
Age										
18–24	5.3 ± 1.07	0.002	4.3 ± 1.00	<0.001	4.6 ± 1.05	<0.001	4.6 ± 1.12	0.05	4.7 ± 0.77	<0.001
25–34	5.4 ± 1.03		4.4 ± 0.97		4.7 ± 0.97		4.6 ± 1.04		4.8 ± 0.76	
35–44	5.6 ± 1.02		4.7 ± 1.07		5.1 ± 0.97		4.9 ± 1.01		5.0 ± 0.78	
45–54	5.7 ± 0.84		4.8 ± 0.95		5.2 ± 0.88		4.9 ± 1.00		5.1 ± 0.70	
≥55	6.1 ± 0.91		5.1 ± 0.98		5.5 ± 0.88		5.2 ± 1.03		5.4 ± 0.78	
Position										
Student	5.3 ± 1.06	0.020	4.3 ± 1.02	<0.001	4.6 ± 1.04	<0.001	4.6 ± 1.11	0.12	4.7 ± 0.77	<0.001
Administrative	5.5 ± 1.08		4.7 ± 0.94		5.0 ± 0.97		4.9 ± 0.97		4.9 ± 0.76	
Academic	5.67 ± 0.93		4.79 ± 1.02		5.12 ± 0.94		4.81 ± 1.08		5.07 ± 0.78	
Marital Status										
Single	5.34 ± 1.07	0.005	4.38 ± 1.00	0.045	4.65 ± 1.05	<0.001	4.63 ± 1.10	0.022	4.71 ± 0.78	<0.001
Married	5.61 ± 0.97		4.57 ± 1.06		5.05 ± 0.95		4.86 ± 1.01		4.98 ± 0.77	

**Table 3:** Univariable effects of age, gender, position, and marital status and their interactions on global EI.

Effect	*F*	*p*-Value	η_p_^2^
Age	4.147	**0.042**	0.008
Gender	0.008	0.929	0.000
Position	1.220	0.296	0.005
Marital status	0.350	0.554	0.001
Age × gender	0.166	0.684	0.000
Age × position	1.993	0.137	0.008
Age × marital status	1.646	0.200	0.003
Gender × position	3.952	**0.020**	0.016
Gender × marital status	0.958	0.328	0.002
Position × marital	3.435	**0.033**	0.014
Age × gender × position	4.364	**0.013**	0.017
Age × gender × marital status	7.127	**0.008**	0.014
Age × position × marital status	2.560	0.078	0.010
Gender × position × marital status	7.175	**0.001**	0.028

Significant effects are shown in bold. The four-way interaction is not displayed because the cell sizes were too small for valid analysis.

## Data Availability

The data presented in this study are available on request from the corresponding author. The data are not publicly available due to ethical constrains.

## Supplementary Materials

The following supporting information can be downloaded at: https://www.mdpi.com/article/10.3390/bs13110911/s1, Table S1: TEIQue-Short Form in English; Table S2: TEIQue-Short Form in Arabic.
