# Supplementary material for: Exploring Emotional Intelligence and Sociodemographics in Higher Education; the Imperative for Skills and Curriculum Development
Source: Behav Sci (Basel). 2023 Nov 8;13(11):911. doi: 10.3390/bs13110911 (PMC10669286; doi:10.3390/bs13110911)
Supplement: Supplementary file 1 [file behavsci-13-00911-s001.zip › behavsci-2657297-supplementary.pdf]

Supplementary Table S1: TEIQue-Short Form in English

TEIQue-SF

Instructions: Please answer each statement below by putting a circle around the number that best reflects your degree of agreement or disagreement with that statement. Do not think too long about the exact meaning of the statements. Work quickly and try to answer as accurately as possible. There are no right or wrong answers. There are seven possible responses to each statement ranging from 'Completely Disagree' (number 1) to 'Completely Agree' (number 7).

1 . . . . 2 . . . . 3 . . . . 4 . . . . 5 . . . . 6 7

Completely Disagree ... **Completely Agree**

1. Expressing my emotions with words is not a problem for me.	1	2	3	4	5	6	7
2. I often find it difficult to see things from another person's viewpoint.	1	2	3	4	5	6	7
3. On the whole, I'm a highly motivated person.	1	2	3	4	5	6	7
4. I usually find it difficult to regulate my emotions.	1	2	3	4	5	6	7
5. I generally don't find life enjoyable.	1	2	3	4	5	6	7
6. I can deal effectively with people.	1	2	3	4	5	6	7
7. I tend to change my mind frequently.	1	2	3	4	5	6	7
8. Many times, I can't figure out what emotion I'm feeling.	1	2	3	4	5	6	7
9. I feel that I have a number of good qualities.	1	2	3	4	5	6	7
10. I often find it difficult to stand up for my rights.	1	2	3	4	5	6	7
11. I'm usually able to influence the way other people feel.	1	2	3	4	5	6	7
12. On the whole, I have a gloomy perspective on most things.	1	2	3	4	5	6	7
13. Those close to me often complain that I don't treat them right.	1	2	3	4	5	6	7
14. I often find it difficult to adjust my life according to the circumstances.	1	2	3	4	5	6	7
15. On the whole, I'm able to deal with stress.	1	2	3	4	5	6	7
16. I often find it difficult to show my affection to those close to me.	1	2	3	4	5	6	7
17. I'm normally able to "get into someone's shoes" and experience their emotions.	1	2	3	4	5	6	7
18. I normally find it difficult to keep myself motivated.	1	2	3	4	5	6	7
19. I'm usually able to find ways to control my emotions when I want to.	1	2	3	4	5	6	7
20. On the whole, I'm pleased with my life.	1	2	3	4	5	6	7
21. I would describe myself as a good negotiator.	1	2	3	4	5	6	7
22. I tend to get involved in things I later wish I could get out of.	1	2	3	4	5	6	7
23. I often pause and think about my feelings.	1	2	3	4	5	6	7
24. I believe I'm full of personal strengths.	1	2	3	4	5	6	7
25. I tend to "back down" even if I know I'm right.	1	2	3	4	5	6	7
26. I don't seem to have any power at all over other people's feelings.	1	2	3	4	5	6	7
27. I generally believe that things will work out fine in my life.	1	2	3	4	5	6	7
28. I find it difficult to bond well even with those close to me.	1	2	3	4	5	6	7
29. Generally, I'm able to adapt to new environments.	1	2	3	4	5	6	7
30. Others admire me for being relaxed.	1	2	3	4	5	6	7

https://psychometriclab.com/

- 6 questions to assess Well-being domain (questions number 5, 9, 12, 20, 24, and 27)
- 6 questions to assess Self-control domain (questions number 4, 7, 15, 19, 22, and 30)
- 8 questions to assess Emotionality domain (questions number 1, 2, 8, 13, 16, 17, 23, and 28)
- 6 questions to assess Sociability domain (questions number 6, 10, 11, 21, 25, and 26)
- 4 questions to assess Adaptability and Self-motivation domains (questions number 3, 14, 18, and 29)

Supplementary Table S2: TEIQue-Short Form in Arabic

TEIQue-SF

التعليمات:

يُرجى الإجابة على كلٍّ من العبارات التالية عبر وضع دائرة حول الرقم الذي يعبّر عن مستوى موافقتك أو رفضك للعبارة، ما من إجابة صحيحة أو خاطئة على هذه الأسئلة. يُرجى الإجابة بشكل سريع وعدم الاسترسال بالتفكير في المعنى الدقيق لكلّ عبارة. يُرجى إعطاء الإجابة الأكثر دقّة على كلّ عبارة. يمكنك الاختيار بين سبع إجابات على مقياس من 1 إلى 7 حيث 1 = لا أوافق أبداً و7 = أوافق تماماً.

1....................2....................3....................4....................5....................6....................7

لا أوافق أبدا ... أوافق تماما

1.	لا أجد صعوبة في التعبير عن عواطفي بالكلام	1	2	3	4	5	6	7
2.	غالباً ما أجد صعوبة في رؤية الأمور من منظار الآخرين	1	2	3	4	5	6	7
3.	بشكل عام، أنا شخص مندفع جداً	1	2	3	4	5	6	7
4.	أجد صعوبة عادةً في تعديل عواطفي	1	2	3	4	5	6	7
5.	لا أجد بشكل عام أنّ الحياة ممتعة	1	2	3	4	5	6	7
6.	أستطيع أن أتعامل بشكل فعّال مع الآخرين	1	2	3	4	5	6	7
7.	أميل إلى تغيير رأيي تكراراً	1	2	3	4	5	6	7
8.	في أوقات عديدة لا يمكنني معرفة العواطف التي أشعر بها	1	2	3	4	5	6	7
9.	أشعر أنه لدي العديد من الصفات الحسنة	1	2	3	4	5	6	7
10.	غالبا ما أجد صعوبة في الوقوف مع حقي	1	2	3	4	5	6	7
11.	أستطيع عادة التأثير على شعور الآخرين	1	2	3	4	5	6	7
12.	بشكل عام تكون نظرتي متشائمة حول معظم الأمور	1	2	3	4	5	6	7
13.	يلومني غالباً الأشخاص المقرّبون لي بأني لا أعاملهم جيّدًا	1	2	3	4	5	6	7
14.	غالباً ما أجد صعوبة في التأقلم حسب الظروف	1	2	3	4	5	6	7
15.	أستطيع بشكل عام أن أتعامل مع التوتّر	1	2	3	4	5	6	7
16.	أجد غالباً صعوبة في التعبير عن عاطفتي للأشخاص المقرّبين منّي	1	2	3	4	5	6	7
17.	عادةً أستطيع أن أضع نفسي في مكان الآخرين وأختبر عواطفهم	1	2	3	4	5	6	7
18.	أجد عادةً صعوبة في أن أحافظ على اندفاعي	1	2	3	4	5	6	7
19.	أستطيع عادةً أن أجد طريقة للسيطرة على عواطفي إذا أردت	1	2	3	4	5	6	7
20.	بشكل عام، أنا راضٍ بحياتي	1	2	3	4	5	6	7
21.	أصف نفسي كمفاوض جيّد	1	2	3	4	5	6	7
22.	أميل عادةً إلى التورط في أمور أتمنى لاحقًا لو يمكنني التخلص منها	1	2	3	4	5	6	7
23.	غالباً ما استوقف نفسي وأفكر بمشاعري	1	2	3	4	5	6	7
24.	أعتقد أنّني أتمتّع بالكثير من نقاط القوّة	1	2	3	4	5	6	7
25.	أميل إلى التراجع عن موقفي حتى عندما أعلم انني محق	1	2	3	4	5	6	7
26.	لا يبدو أنه لدي أي تأثير على مشاعر الآخرين بأي شكل	1	2	3	4	5	6	7
27.	أنا أعتقد عموماً بأنّ الأمور ستكون لصالحي في حياتي	1	2	3	4	5	6	7
28.	أجد صعوبة حتى في التواصل مع المقربين مني	1	2	3	4	5	6	7
29.	في شكل عام، أستطيع أن أتأقلم في بيئات جديدة	1	2	3	4	5	6	7
30.	يعجب الآخرون بهدوئي	1	2	3	4	5	6	7

https://psychometriclab.com/
